# The pathogenicity of variant c.597dup in *SLC19A3* and treatability of its phenotype remain unconfirmed

**DOI:** 10.1055/s-0044-1786024

**Published:** 2024-04-12

**Authors:** Josef Finsterer, Fulvio A. Scorza

**Affiliations:** 1 Neurology & Neurophysiology Center, Vienna, Austria.; 2 Universiade Federal de São Paulo, Escola Paulista de Medicina, São Paulo SP, Brazil.

Dear Editor,

We read with interest the article by Freitas et al. about a two-month-old male with Leigh syndrome due to the variant c.597dup in *SCL19A3* . 1 Cerebral MRI showed a symmetrical mixture of cytotoxic and vasogenic oedema in the midbrain, superior vermis, cerebellar hemispheres, genu corporis callosum, internal capsules, thalamus, and basal ganglia (putamen), occipital cystic lesions, and spectroscopy a lactate peak. 1 Despite administration of thiamine and biotin, the patient apparently died. 1 The study is impressive, but some points require discussion.

We do not believe that the mutated gene is the “ *SCL19A3* ” gene as stated in the title and text. 1 If the authors mean the “ *SLC19A3* ” gene, this should be corrected.

We disagree with the statement that Leigh syndrome due to variants in *SLC19A3* is potentially treatable. 1 Although the gene encodes the thiamine-transport-2, 2 previously reported pediatric cases did not show a positive treatment response, like the index case. In a 4 months-old patient with infantile Leigh-like syndrome due to the variant c.91dupT in *SLC19A3,* high doses of thiamine (150mg/d) and biotin (40mg/d) were ineffective. 2 The patient died four months after birth. In this context, the exact dosages of thiamine and biotin administered to the index patient are missing. The two cases described by Debs et al. who responded to biotin were adults (33 and 29 years old) and had a completely different phenotype. 3 Treatment response is probably related to the age of onset. 4

We disagree that the variant was the cause. Mutations in *SLC19A3* usually lead to autosomal recessive inherited neurodegenerative disorders with broad phenotypic heterogeneity. 2 However, the variant reported in the index patient occurred in a heterozygous form, suggesting autosomal dominant transmission. 1 Autosomal dominant transmission implies that 50% of the offspring are phenotypically affected. However, it is conceivable that the second allele carried a copy number variant (CNV) or that the phenotype was also due to an intronic variant that cannot be detected by WES.

We should know whether the variant occurred sporadically in the index patient or was inherited. In the case of inheritance, it should be reported whether parents or other first-degree relatives also carried the variant and were phenotypically affected. Before the phenotype of the index patient can be attributed to the *SLC19A3* variant, functional and biochemical studies must be performed to demonstrate its pathogenicity.

In summary, before drawing final conclusions from the presented case, clinical genetic testing of the parents and other first-degree relatives should be performed and the pathogenicity of the variant should be confirmed. A fatal infantile disease should not be described as “potentially treatable”.
